# 

**Published:** 1918-04-27

**Authors:** 


Rates of Subscription: Twelve months, 6/6; Six months, 4/-; three months, 2/-, post free to any address in the United Kingdom. Abroad, Twelve months, 8/8; Six months, 5/-; Three months, 2/6, post free. **THE HOSPITAL "Index" to Volume LXII, April 1917 to September 1917, is now ready, price 2½d. per copy, post free.** Address The Manager, The Hospital, "The Hospital" Building, 28/29 Southampton Street, Strand, London, W.C. 2.

### Matrons' and Sisters' Appointments.

Ladywell Sanatorium, Salford.—Miss G. Luke has been appointed sister. She was trained at Mile End Infirmary, has been sister at Townleys Hospital, Bolton; Joyce Green Hospital, Dartford; City Hospital, Bristol; Western Fever Hospital, Fulham; and at the Military Hospital, Griffithstown, Mon.

Red Cross Hospital, Sutton, Surrey.—Miss Vera Diss has been appointed sister. She was trained at the Willesden Infirmary, and has been staff nurse at the Military Hospital, Boscombe. She has also done private nursing.

Royal Hospital for Sick Children, Aberdeen.—Miss Marion Kennedy has been appointed surgical sister. She was trained at Oldham Royal Infirmary, where she has been sister and night sister.

Royal Samaritan Hospital for Women, Glasgow.—Miss S. A. Christie has been appointed sister. She was trained at Bolton General Infirmary and Dispensary and at Lennox Joint Hospital, Milton of Campsie. She has been casualty sister at the Borough Hospital, Birkenhead, and has done private nursing.

### NEWMAN'S FORTREVIVER. A LIQUEUR TONIC.

(H. and C. Newman, 41 and 42 Upper Rathbone Place, London, W. 1.)

We have received a sample of this preparation. It is a vegetable tonic which has the appearance and to some extent the taste of wine, but is non-alcoholic. Its taste is pleasant, slightly bitter, and slightly astringent. It may be taken as such in doses of about a port wine glassful three times a day between meals, or diluted with soda-water or milk to taste, about a port wine glassful to the tumbler of diluent being an average proportion. It can also be taken with wine or with whisky or with brandy-and-soda, and it is claimed for it that it improves the taste of hock- or claret-cup. Its medicinal properties depend to some extent upon its gastronomic ones, and also upon its actual constituents, the most important of these latter being those contained in fruit juices and vegetable iron compounds. In our opinion, as a domestic tonic, appetiser, or refreshing drink within the limitations applicable to such preparations, it is to be recommended, and is likely to obtain a considerable sphere of usefulness.

### Ramsgate General Hospital.

In our issue of April 20 (p. 45) the name of the private ward at the above hospital should have been stated as the Dame Janet Stancomb-Wills Ward, and the Women's bed founded by Miss Stancomb-Wills as the Lady Wills of Blagdon Bed.

### Forthcoming Events.

### The Feeding and Care of Infants.

A series of lectures and demonstrations by Dr. Truby King, C.M.G., will be given on Friday, May 3, and succeeding Tuesdays and Fridays in May, at 5.30 p.m., in the Governors' Hall of St. Thomas's Hospital. (See under Institutional News, p. 72.)

